# Study of Hybrid Neurofuzzy Inference System for Forecasting Flood Event Vulnerability in Indonesia

**DOI:** 10.1155/2019/6203510

**Published:** 2019-02-25

**Authors:** Sri Supatmi, Rongtao Hou, Irfan Dwiguna Sumitra

**Affiliations:** ^1^School of Computer and Software, Nanjing University of Information Science and Technology, No. 219 Ningliu Road, Pukou, Nanjing, Jiangsu 210044, China; ^2^Computer Engineering Department, Universitas Komputer Indonesia, No. 102-116 Dipati Ukur, Bandung, West Java 40132, Indonesia; ^3^Postgraduate of Information System Department, Universitas Komputer Indonesia, No. 102-116 Dipati Ukur, Bandung, West Java 40132, Indonesia

## Abstract

An experimental investigation was conducted to explore the fundamental difference among the Mamdani fuzzy inference system (FIS), Takagi–Sugeno FIS, and the proposed flood forecasting model, known as hybrid neurofuzzy inference system (HN-FIS). The study aims finding which approach gives the best performance for forecasting flood vulnerability. Due to the importance of forecasting flood event vulnerability, the Mamdani FIS, Sugeno FIS, and proposed models are compared using trapezoidal-type membership functions (MFs). The fuzzy inference systems and proposed model were used to predict the data time series from 2008 to 2012 for 31 subdistricts in Bandung, West Java Province, Indonesia. Our research results showed that the proposed model has a flood vulnerability forecasting accuracy of more than 96% with the lowest errors compared to the existing models.

## 1. Introduction

Flood disaster [1] is one of the significant problems in some countries including Indonesia. Flood disaster occurs mostly in populated areas. The high rainfall in many cities increases the risk of flooding. The flood occurs mainly because of a high rate of rainfall for extended periods in the wet season. The drainage system could not control the problem of rising water volume in a river or a channel. The critical thing in meteorology, hydrology (e.g., flood warning), environmental policy, and agriculture, is the accurate measurement of rainfall [2]. As a result of flood, people loss their homes and their crops, miss the school education, and even worse, lose their life.

In October 2016, some regions in Indonesia were affected by the flood, and the disaster management officials reported that the flash flood in the Bandung city caused death of one person and damaged thousands of homes. The National Disaster Management Authority, also known as BNPB, informed that there was 77 mm of rainfall in the town in just 1.5 hours around midday [3, 4]. Most of the areas of the city were inundated with flood water between 120 cm and 200 cm deep [3, 4]. In the same month, a part of Gorontalo, Indonesia, was affected by flood; at least 1,500 homes were damaged, and around 4,500 people were forced to evacuate their homes [5, 6]. In December 2016, a part of West Nusa Tenggara Province, Indonesia, was hit by two floods in the space of days, forcing over 100,000 people to leave their homes. BNPB informed that thousands of homes were damaged, and streets were left with flood water from one meter to three meters deep [4, 6]. On January 26, 2017, flood hit North Sulawesi, Indonesia. This flood affected 11 villages in Gorontalo Utara and damaged over 700 homes, some schools, and agricultural land, forcing more than 100 people to leave their places [4, 7]. On March 3, 2017, at least six people died, two seriously injured, and thousands displaced due to floods in Indonesia's West Sumatra Province. The flood also caused power and communication outage, and over one hundred electrical substations were shut down, leaving almost 15,000 houses without electricity [4, 8].

The answer to overcome the flood disaster by applying the fuzzy system is assisting, predicting, and deciding these events. In 1965, Lotfi, a mathematician who created the theory of fuzzy logic, derived the result of the insufficiency of Boolean algebra for many real-world problems [9, 10]. In fact, as most of the information is imprecise, one of the human's greatest abilities is to process inaccurate problems and vague information efficiently. The *Oxford English Dictionary* defined the word “Fuzzy” as blurred, indistinct, imprecisely defined, confused, or vague. The fuzzy systems are knowledge-based or rule-based systems. The heart of a fuzzy system is the so-called knowledge-based fuzzy IF-THEN rules [11].

Many researchers have argued on meteorological forecasting, with the purpose to help assessing the disaster impact in some regions. Forecasting is also used in other areas such as detection and prediction of diseases [12, 13]. Asklany et al. [14] proposed the probabilistic prediction using two skill scores, namely, the Brier Score and Friction Score. Those researchers mentioned that the rainfall event prediction was highly accurate with the data aggregated by the stations with increasing output toward the real-time event. Otherwise, the fuzzy inference system output precedes the recorded maximum data in six hours before the rainfall. Li-Chiu et al. [15] introduced a model to predict the stream-flow an hour after a flood event occurs. They also compared the result using a fuzzy exemplar-based inference system with backpropagation neural network to show that a fuzzy model performs better than the neural network. de Bryun et al. [16] described a method based on fuzzy arithmetic to estimate the possible range of flow rates and water level based on possible rainfall events by the forcing and uncertainty model. A previous research [17] explained a rainfall level detection system to predict the weather using the Mamdani method. In [18], the rainfall event was analyzed using the defuzzification method. In [19], the genetic algorithm (GA) was discussed to obtain a higher accuracy of the rainfall forecasting system. In [20], the agricultural irrigation control using the fuzzy method for determining water quantities was studied. In [21], the Mamdani FIS was studied to predict the rainfall events in the Khorasan region. In [22], flood events were predicted using numerical weather prediction (NWP) with improved accuracy. In [23], the main objective is to predict the high-risk area by water level using Artificial Neural Network in Masantol, Pampanga. In [24], a flood event prediction model based on SVM and boosting algorithm was presented. Mitra et al. [25] explained that flood forecasting employs the Internet of Things and artificial neural networks based on the water level in a river basin. In [26], the flood forecasting system development in the upper reaches of the Zhangweihe River Basin was discussed. In [27], the grey model for flood forecasting based on the rainfall and the flood index in Nanjing, China, was studied. All the previous studies described flood forecasting based on different methods and employed rainfall parameters. Gilbert Brunet described meteorological forecasting using numerical weather prediction (NWP) and obtained results with good quality and accuracy with the complexity of numerical computing; however, a certain percentage for the accuracy was not obtained [28]. Mencattini et al. utilized the type-2 fuzzy logic system in their system which aims at meteorological forecasting. The measurement consists of humidity and temperature that was obtained from the Neuronica Lab at Politecnico of Torino (Italy). This research achieved accurate forecasting results even many hours in advance with a mean absolute error of 33.8% for humidity and 8.2% for temperature [29]. Hansen and Riordan employed case-base reasoning (KNN method) and fuzzy set theory to predict the airport weather. The data tested were more than 300,000 hours of airport weather observation recorded for 36 years. The testing methods used to solve the problem were cloud ceiling and visibility of those airports produced by 6-hour predictions. The result showed good accuracy for the airport weather prediction [30]. Ding et al. employed the artificial neurofuzzy inference system (ANFIS) and clustering technique. The ANFIS was used to make sure of the minimal errors of the parameters of membership functions (MFs) and the design of MFs. The result was efficient and close to that of actual load forecasting in practice. However, the result had drawbacks of the neural network and was sensitive to the noise [31]. Mountis and Levermore utilized the fuzzy logic, artificial neural network (ANN), and neurofuzzy method to forecast the weather at the Manchester International Airport for the years 1982–1995, which examined only summer data. The accuracy of the forecast depends on how well its parameters are defined and adjusted by the ANN method. The results of this research provided satisfactory MAE values: neurofuzzy 17 %, ANN 18%, and fuzzy logic 30% [32]. Ahmad et al. in their research employed the ANN method with the supervised learning technique for forecasting. The study area in this research was Senai, Johor, Malaysia. Data inputs for the forecasting were pressure, time of day, dry bulb temperature, wet bulb temperature, dew point, wind speed, wind direction, cloud cover, and rainfall in the past hour. The accuracy was 78% [33]. Qin et al. employed ANNs with a gradient descent technique RPROP with dynamic tunneling technique to train the actual data of past 24 months in 1999–2000 with the data tested of six months (2001/1–2001/3, 2001/7–2001/9) from Chongqing, China. The result gave faster convergence and global optimization for forecasting. The result is close to the real data [34]. In [35], the flood prediction employing artificial neural network was described, and the flood water level was successfully predicted 24 hours, 48 hours, and 72 hours ahead of time. The results showed MAE varying from 0.6 to 0.9 and RMSE varying from 0.05 to 0.11. In 2012, in a study case in Tancheon, South Korea, Choi et al. employed neurofuzzy system to forecast the flood [36]. The results showed the average RMSE was 0.367%.

This study proposes a hybrid approach based on the neural network and fuzzy inference system for flood event vulnerability, namely, hybrid neurofuzzy inference system (HN-FIS). The HN-FIS is a model which can automatically learn and also obtain the output which can present the essence of fuzzy logic. The system was applied in 31 subdistricts in Bandung. The flood forecasting depends on several variable inputs: population density, altitude of the area, and rainfall in time series from 2008 to 2012. The main contributions of this paper are (i) presenting a hybrid forecasting for flood vulnerability based on the neural network and fuzzy inference system for accurate flood forecasting employing data variables which utilized Bandung database for flood vulnerability forecasting and (ii) developing an effective hybrid forecasting approach for flood vulnerability with higher accuracy.

## 2. Study Area

**Figure 1:**
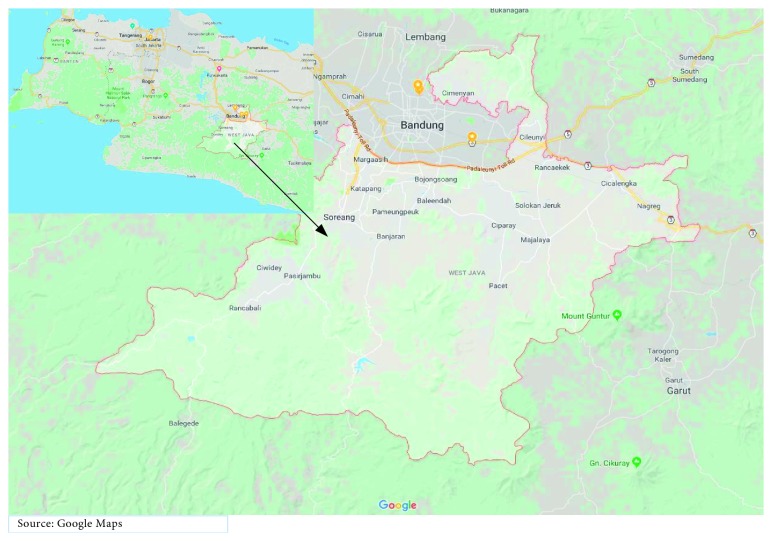
Study area map of Bandung, West Java, Indonesia.

This study used data collected from Bandung, West Java Province, Indonesia (Figure 1). Geographical conditions of the subdistricts have total area cover about 176238.67 Ha, which lies between longitudes 107022′ and 108050′ east and latitudes 6041′ and 7019′ south. Most of the area of Bandung is located between the surrounding hills and mountains. To the north lies Mount Bukittunggul with a height of 2200 meters and Mount Tangkuban Perahu with a height of 2076 meters, which borders West Bandung and Purwakarta. On the south, there is Mount Patuha with a height of 2249 meters, Mount Malabar with a height of 2321 meters, Mount Papandayan with a height of 2262 meters, and Mount Guntur with a height of 2249 meters. Bandung has mountainous areas with an average slope of from 0–8%, 8–15%, to above 45%. Bandung has a tropical climate that is influenced by the monsoon climate with average rainfall between 2000 mm and 3000 mm per year (Table 1). Air temperature ranges from 12°C to 24°C, which results in air humidity of about 78% in the rainy monsoon season and 70% in the hot dry season.

The primary variables (Tables 1–3 used to obtain the flood forecasting value were divided into four parameters (population density, altitude of the area, rainfall, and vulnerability of flood). The definitions of the main parameters and the fuzzy value are presented below:

1. Population density: the population density of the subdistricts in which the people are located. The value zero means the population density is very low (less than or equal to 50 persons/km^2^). The value one means the population density is very high (that is, greater than 400 persons per square kilometer).
2. Altitude of the area: the distance above sea level of the land, mountain, sea bed, or any other place. If the altitude of an area is less than 200 meters above sea level or coastal area, it is shown in fuzzy as low level (value 0), and the altitude of area greater than 350 meters above sea level or mountain area means the altitude in high level (value 1).
3. Rainfall: according to the rate of rainfall, it is classified as low level “0,” light rain which happens when the precipitation rate is less than 20 mm per hour, and high level “1,” very heavy rain which happens when the precipitation rate is more than 100 mm per hour.
4. Vulnerability of flood: the inability to resist a flood or to respond when the flood occurs: 0 = safe (when the area is secured from flood) and 1 = danger (when the area is under a threat of flood).

## 3. Material Parameters

**Figure 2:**
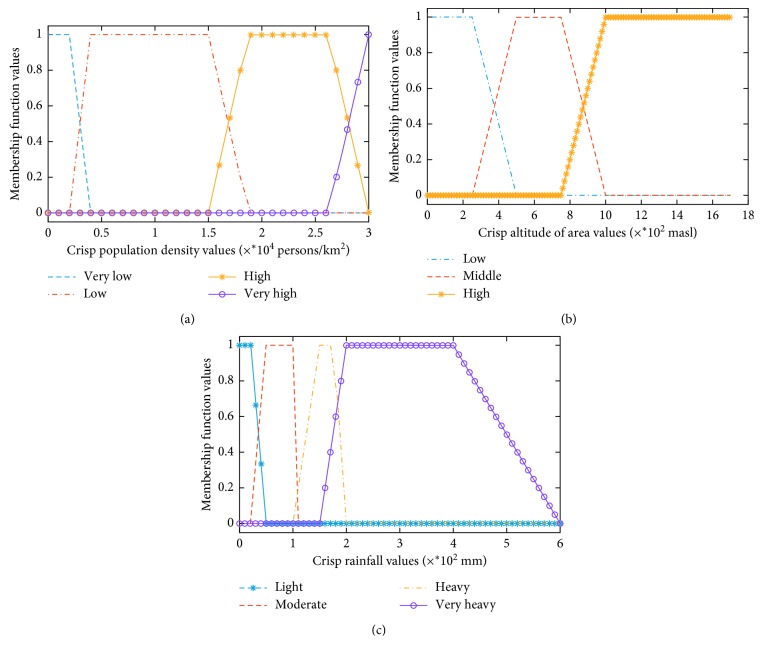
Membership function curves of flood forecasting fuzzy variable premises.

**Figure 3:**
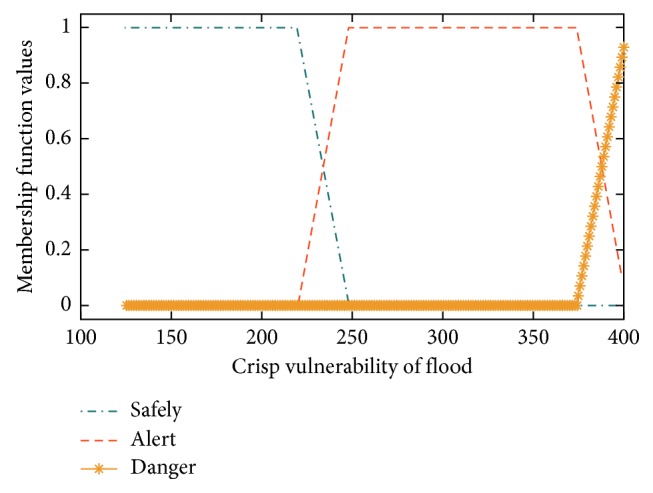
Membership function curves of flood forecasting fuzzy variable output.

The flood vulnerability in 31 subdistricts in Bandung is predicted using the Mamdani system, Sugeno system, and proposed flood forecasting model (HN-FIS). It consists of three inputs for vulnerability of flood level: population density, altitude of the area, and rainfall. The population density is in the range of 350 to 9000 people/km^2^. The altitude of the area is in the range of 0 to more than 1000 meters above sea level (masl). The rainfall is in the range of 0 to more than 200 mm. All the fuzzy models in this research were applied in the trapezoidal type trying to find the best one for the prediction of the vulnerability of flood event. The classification of fuzzy sets employed in the flood forecasting method is presented in Table 4. There are three or four indexes to indicate the parameters according to Tables 1–3, respectively. The inputs have three or four membership functions as shown in Tables 5–7 presented for the population density, altitude of the area, and rainfall (also shown in Figures 2(a)–2(c)), respectively. The output (the vulnerability of flood) is taken in values ranging from 0 to more than 374, presented for three conditions (safe, alert, and danger) as shown in Table 8 and Figure 3.

According to Table 5, the population density has four fuzzy classifications such as very low, low, high, and overpopulation, respectively (Table 2).

Table 6 describes the fuzzy classification for the altitude of the three-level fuzzy area parameters, such as low, moderate, and high.

In Table 7, the fuzzy classification was divided into four levels such as low, moderate, high, and extreme rainfall.

Table 8 provides the fuzzy classification for the output of the vulnerability of flood. It has three levels fuzzy: safe, alert, and danger.

## 4. Distributed Implementation of Hybrid Flood Forecasting Model

Based on the measurement and theoretical analysis, both Mamdani and Sugeno models required a significant number of forecasting to obtain a higher level of accuracy for the vulnerability of flood. The models considered the parameters that are in flood forecasting. We present the Mamdani model and the Sugeno model for practically distributed flood prediction.

### 4.1. Mamdani Fuzzy Inference System (Mamdani FIS)

**Figure 4:**
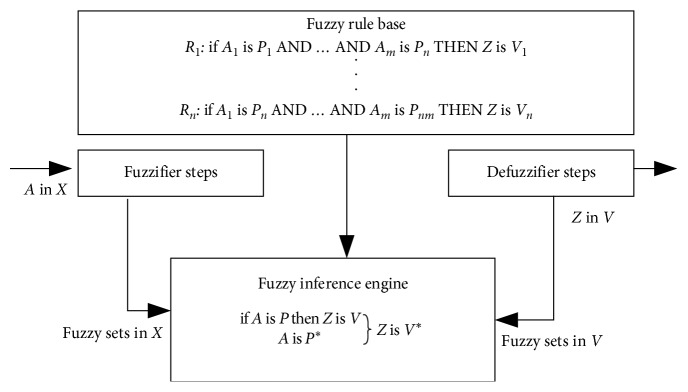
Mamdani fuzzy inference system architecture.

Mamdani and Assilian proposed the first type of fuzzy inference system (FIS) in 1975 [37]. A Mamdani FIS has fuzzy inputs and fuzzy output. The architecture of the Mamdani FIS to show the mapping from input space into output space can be seen in Figure 4 (referred from [38]).

According to Figure 4, the system of the crisp input source is first transformed by *fuzzifier* into a set of linguistic variables in *X*. The *fuzzy inference engine* using the input variables and the rules to decide on the *fuzzy rule base* derives a set of conclusions in *V*. *Defuzzifier* purpose to convert into a crisp number which corresponds to the output of the system [39].

### 4.2. Sugeno Fuzzy Inference System (Sugeno FIS)

Takagi and Sugeno proposed the first fuzzy inference system, namely Sugeno FIS, in 1985 [40] and by Sugeno and Kang in 1988 [41]. A Sugeno FIS has fuzzy inputs and a crisp output.

**Figure 5:**
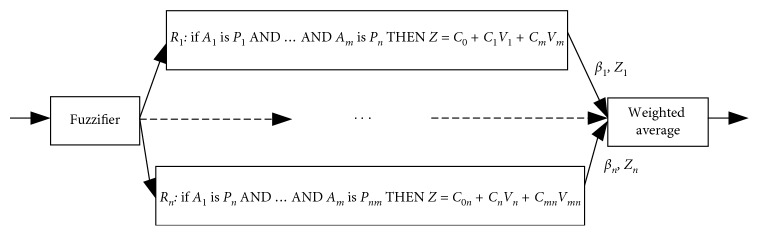
The architecture of the Sugeno fuzzy inference system.

Referring to the same assumptions as for the Mamdani FIS, the architecture for the Sugeno FIS is illustrated in Figure 5 (according to [38]).

In this short of fuzzy inference system, only the antecedents of the rules are fuzzy, and it means the rules act as an inference mechanism themselves [38, 41]. The main difference of this architecture which compared with Mamdani FIS is that the Sugeno FIS does not require a defuzzification to obtain a crisp result output from the rules consequents. The crisp result can be obtained employing a weighted average of the rules crisp consequents using the firing strength level as weights [38, 41, 42].

### 4.3. Proposed Flood Forecasting Model

Takagi and Sugeno [40] presented an adaptive neurofuzzy inference system that was obtained from the neural network and fuzzy logic [43] by catching the advantages of both in one framework. The neural network has the capability of automatic learning. However, this model cannot describe how it acquires the output from decision making. On the other hand, the fuzzy logic can obtain output out of the fuzzy logic decision. However, it does not have the ability of learning automatically [44]. Combining neural network and fuzzy logic can generate input and output data pairs, and it has been successfully used in diverse fields at solving nonlinear issues and indicating problems [45]. In this study, the Sugeno fuzzy multilayer, which is equivalent to a neural network, and the Mamdani fuzzy inference system were combined to form a hybrid neurofuzzy inference system (HN-FIS). The advantage of the proposed model is its capability of automatically learning and obtaining an output of fuzzy logic decision more clearly, which can exhibit human judgment reasonably.

Considering Figures 4 and 5 have the same rule base and fuzzification for the variables, there are several defuzzifiers which can be chosen for a Mamdani FIS that originate similar results in a Sugeno FIS, which means an inevitable overlap between both types of systems. The Mamdani FIS is more widely used, particularly for decision support applications, and mostly refers to the intuitive and interpretability nature of the rule base. On the other hand, the Sugeno FIS do not have a linguistic term, and this interpretability is partially lost [41, 46]. However, since Sugeno FIS rule's consequents can have as many parameters per rule as input values, this translates into more degrees of freedom in its design than a Mamdani FIS, thus providing more flexibility [41]. Mendel reaches this conclusion by comparing the number of possible design parameters for both Mamdani FIS and Sugeno FIS for certain choices of input and output variables [41].

According to that fuzzy inference system, many parameters can be employed in the consequents of the rules of a Sugeno FIS which reasonably approximates a Mamdani FIS. This session described how the proposed flood forecasting model (hybrid neurofuzzy inference system (HN-FIS)) works.

The Takagi-Sugeno (Sugeno) fuzzy model and Mamdani fuzzy model are two great fuzzy rule-based inference systems. The Sugeno fuzzy inference system works well with linear techniques and guarantees continuity of the output surface [40, 47]. However, the Sugeno fuzzy model has difficulties in dealing with the multiparameter synthetic evaluation. It has difficulties in assigning weight to each input and fuzzy rule. The Mamdani fuzzy model has had some advantages such as its intuitive, widespread acceptance, and well suitable to human cognition [37, 48, 49]. The researchers employed the Mamdani model and the Sugeno model as a proposed flood forecasting model (hybrid neurofuzzy inference system), which shows the advantages of those models in the output statement, which is more readibility and easy to understand even by the layperson.

A function needs to be assigned to specify the operation of the Mamdani fuzzy model entirely with the following steps:

1. Operator OR or operator AND to the rule firing strength computation with OR'ed or AND'ed references
2. Consequent membership function calculated from the implication operator based on a given firing strength
3. Aggregate operator used to produce overall output membership function by combining the aggregated qualified consequents
4. Defuzzification operator aims to transform an output membership function to a crisp single output value

If the first step is the AND operator, the second step is a product, the third step is the sum, and the fourth step is the centroid of the area (COA) [50, 51], we can derive the following equations. The advantage of applying hybrid neurofuzzy inference system (HN-FIS) model is the ability of learning because of differentiability during computation.

Equations (1) and (2) [37] provide the sum-product composition. The final crisp output when using centroid defuzzification is equal to the weighted average of the centroid of consequent membership functions:

(1) $$\begin{array}{c} \delta \left(r_{i}\right)=w\left(r_{i}\right)\times \alpha , \end{array}$$

where *δ*(*r*_*i*_) is the weighted factor of *r*_*i*_; *r*_*i*_ is the *i*th fuzzy rule; *w*(*r*_*i*_) is the firing strength of *r*_*i*_; and *α* is the area of the consequent membership functions of *r*_*i*_.

(2) $$\begin{array}{c} Z_{\text{COA}}=\frac{\int_{Z_{i=1}}^{n}\mu B^{\prime }\left(z\right)z\;\;dz}{\int_{Z_{i=1}}^{n}\mu B^{\prime }\left(z\right)\;\;dz}=\frac{w_{1}\alpha_{1}z_{1}+\ldots +w_{n}\alpha_{n}z_{n}}{w_{1}\alpha_{1}+\ldots +w_{n}\alpha_{n}}, \end{array}$$

where *α*_*i*_ and *z*_*i*_ are the area and the center of the consequent membership function, *μB*_*i*_(*z*), respectively.

The rules of the HN-FIS model are given as follows:

  Rule 1(*r*_*1*_): if *P* is *P*_*1*_, *A* is *A*_*1*_, and *R* is *R*_1_, then *Z* *=* *B*_*1*_.
  Rule 2 (*r*_*2*_): if *P* is *P*_*2*_, *A* is *A*_*2*_, and *R* is *R*_2_, then *Z* *=* *B*_*2*_.
  …
  Rule *n*(*r*_*n*_): if *P* is *P*_*n*_*A* is *A*_*n*_ and *R*_f_ is R_fn_, then *Z* *=* *B*_*n*_.

**Figure 6:**
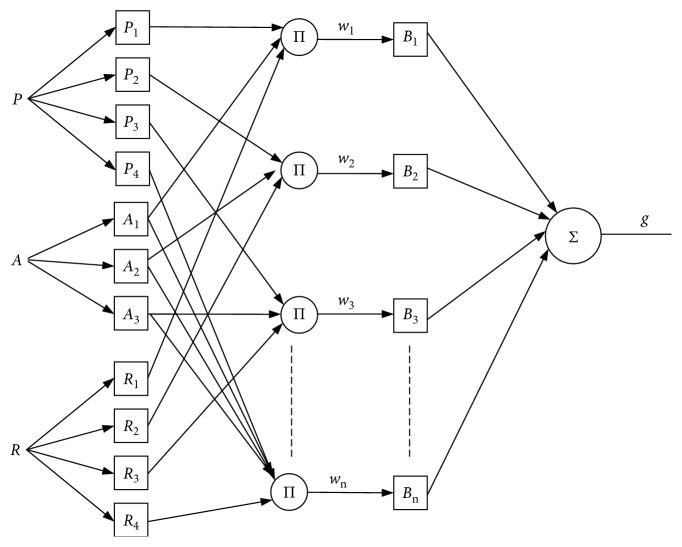
Proposed flood forecasting model (HN-FIS) architecture.

According to the rules, the HN-FIS model can be expressed as shown in Figure 6.

*P*, A, and *R* represent the inputs, which are population density (Table 5), altitude of the area (Table 6), and rainfall (Table 7). P_1,_ P_2,_ P_3_, and P_4_ represent the membership functions of population density. *A*_1,_*A*_2,_ and *A*_3_ represent the membership functions of the altitude of the area. *R*_1,_*R*_2,_*R*_3_, and *R*_4_ represent the membership functions of rainfall. The firing strength denoted as *w*_1_, *w*_2_,…, *w*_*n*_. *B*_1_, *B*_2_,…, *B*_*n*_ represents the following parameters which need to be adjusted. The consequent parameter B is a multiplication of *α*_*i*_ and *z*_*i*_ (according to (2)). The membership function of the vulnerability output is denoted as *g*.

The HN-FIS architecture is composed of five layers, and Figure 6 illustrates the output of each layer.

**First layer:** fuzzification layer
  (3) $$\begin{array}{c} L_{1,i}=\mu P_{i}\left(P\right),\;\;i=1,2,3,4,\\ L_{1,i}=\mu A_{i-4}\left(A\right),\;i=5,6,7,\\ L_{1,i}=\mu R_{i-7}\left(R\right),\;i=8,9,10,11. \end{array}$$

The membership function is the generalized trapezoidal function, denoted as follows:

(4) $$\begin{array}{c} \mu P_{i}\left(P\right)=\left\{\begin{array}{c} \begin{array}{cc} 0, & P<s_{i},\\ \frac{P-s_{i}}{t_{i}-s_{i}}, & \;s_{i}\leq P\leq t_{i}, \end{array}\\ \begin{array}{cc} 1, & \;\;t_{i}\leq P\leq u_{i},\\ \frac{v_{i}-P}{v_{i}-u_{i}}, & \;u_{i}\leq P\leq v_{i},\\ 0, & v_{i}\leq P, \end{array} \end{array}\right.\\ \mu A_{i}\left(A\right)=\left\{\begin{array}{c} \begin{array}{cc} 0, & A<s_{i}\\ \frac{A-s_{i}}{t_{i}-s_{i}}, & \;\;\;s_{i}\leq A\leq t_{i}, \end{array}\\ \begin{array}{cc} 1, & t_{i}\leq A\leq u_{i}\\ \frac{v_{i}-A}{v_{i}-u_{i}}, & \;\;\;u_{i}\leq A\leq v_{i},\\ 0, & \;\;v_{i}\leq A, \end{array} \end{array}\right.\\ \mu R_{i}\left(R\right)=\left\{\begin{array}{c} \begin{array}{cc} 0,\;\; & R<s_{i},\\ \frac{R-s_{i}}{u_{i}-s_{i}}, & \;s_{i}\leq R\leq u_{i}, \end{array}\\ \begin{array}{cc} 1, & \;\;t_{i}\leq R\leq u_{i},\\ \frac{v_{i}-R}{v_{i}-u_{i}}, & \;u_{i}\leq R\leq v_{i},\\ 0, & v_{i}\leq R. \end{array} \end{array}\right. \end{array}$$

*P* is the crisp value (real value) of population density, *A* is the crisp value of altitude of the area, and *R* is the crisp value of rainfall. {*s*_*i*_*, t*_*i*_*, u*_*i*_,*v*_*i*_} is the premise parameter or the set parameter which is used to denote the membership functions in this model.

 
**Second layer**: the layer of rule or the layer of inference

(5) $$\begin{array}{c} L_{2,i}=w_{i}=\mu P_{i}\left(P\right)\;\;x\;\;\mu A_{i}\left(A\right)\;\;x\;\;\mu R_{i}\left(R\right),\;\;\;i=1,\;\;2,\;\;3,\;\;4. \end{array}$$

In this layer, the product method is generated for the firing strength *w*_*i*_.

**Third layer:** implication layer
  (6) $$\begin{array}{c} L_{3,i}=w_{i}\;\circ \;B_{i},\;\;\;i=1,\;\;2,\;\;3,\;\;\ldots ,\;\;48. \end{array}$$

The product of this layer comes from the implication operator.

**Fourth layer:** aggregation layer
  (7) $$\begin{array}{c} L_{4,i}=O_{1}=\overset{n=48}{\underset{i=1}{\sum }}w_{i}\;\circ \;B_{i},\;\;\;i=1,\;\;2,\;\;3,\;\;\ldots ,\;\;48. \end{array}$$

The result of this layer is the sum of all implication operators in the implication layer. The following parameters are denoted by *B*_i_.

**Fifth layer:** defuzzification layer
  (8) $$\begin{array}{c} L_{5}=O_{2}=g=D\;\circ \;O_{1}. \end{array}$$

The defuzzification (*D*) method and center of the area (COA) were achieved to produce the crisp output *g*.

In this paper, the trapezoidal functions generalized were used for the type of membership functions (MFs) of the inputs and had four nonlinear parameters to be adjusted ({*s*_*i*_*, t*_*i*_*, u*_*i*_*, v*_*i*_}). The MFs of population density have four nonlinear parameters (shown in Table 5 and Figure 2(a)). The MFs of the altitude of the area have three nonlinear parameters (shown in Table 6 and Figure 2(b)). The MFs of rainfall have four nonlinear parameters (shown in Table 7 and Figure 2(c)). In this model, premise parameters are 48, and following parameters are 96. Hence, the total number of the nonlinear parameter is 140.

### 4.4. Evaluation Criteria for Model Performance

If *M*_*i*_ is the measured value for the number of subdistricts and *P*_*i*_ is the prediction value in the same subdistricts, then the error (*E*_*i*_) is defined as

(9) $$\begin{array}{c} E_{i}=M_{i}-P_{i}. \end{array}$$

Since there are measured values and predictions for *n* subdistricts, there will be *n* error terms, and the standard statistical measures can be defined as follows.

*Mean absolute error (MAE)* is the average of all absolute errors, meaning the amount of all absolute errors divided by the number of errors. The equation of MAE is as follows:

(10) $$\begin{array}{c} \text{MAE}\;\;=\frac{1}{n}\sqrt{\overset{n}{\underset{i=1}{\sum }}\left|E_{i}\right|}\times 100\%. \end{array}$$

*Root mean square error (RMSE)* is the square root of the average of squared differences between the measured value and the predicted value shown in the following equation:

(11) $$\begin{array}{c} \text{RMSE}\;\;=\frac{1}{n}\sqrt{\overset{n}{\underset{i=1}{\sum }}{\left|E_{i}\right|}^{2}}\times 100\%. \end{array}$$

The mean absolute error is defined by first making each error positive by taking its absolute value and then averaging the result in the square root. The RMSE is defined by the similar idea of the mean absolute error. In RMSE, the errors are made positive by squaring each one, and then the squared root errors are averaged. The MAE has the advantage of being more interpretable and easier to describe nonspecialists. The RMSE has the advantage of being easier to handle mathematical problems. Each of these statistics deals with measures of accuracy whose size depends on the scale of the data [32, 52].

## 5. Results and Discussion

The discussion of the results begins with explanation of the performance of the proposed vulnerability of the flood forecasting model based on the neurofuzzy system approach, namely HN-FIS. The flood forecasting models are developed employing MATLAB 2017 software [53]. The results are presented as follows.

**Figure 7:**
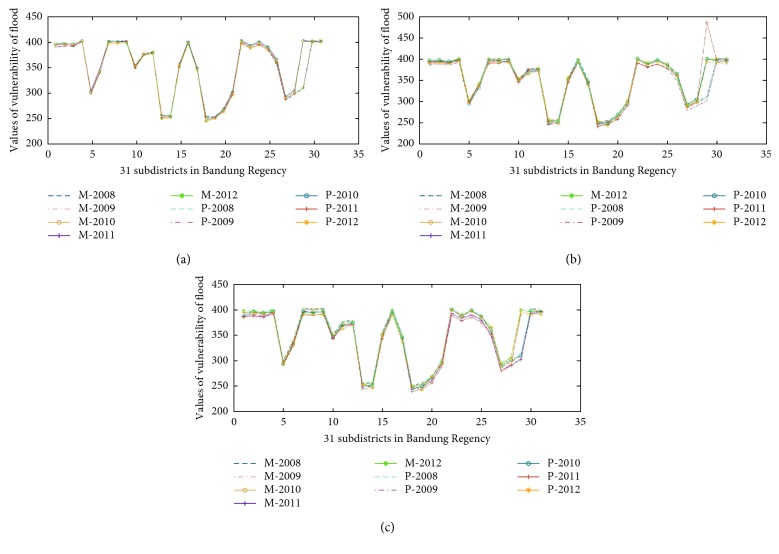
Vulnerability values of flood: (a) proposed model (HN-FIS), (b) Mamdani model, and (c) Sugeno model in 2008, 2009, 2010, 2011, and 2012, respectively.

**Figure 8:**
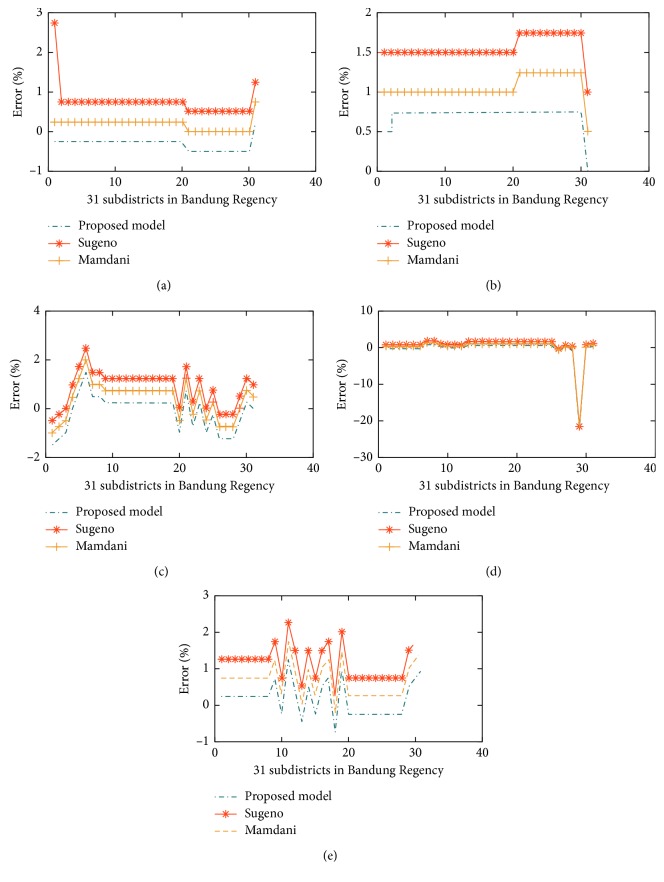
Comparing error of the Mamdani model, Sugeno model, and proposed model (HN-FIS) in (a) 2008, (b) 2009, (c) 2010, (d) 2011, and (e) 2012.

In our experiment, three graphics illustrated in Figure 7 are the result of the vulnerability of flood forecasting employing three models: Mamdani, Sugeno, and HN-FIS, respectively, applied in 31 subdistricts in Bandung, Indonesia. Those models have used to design of experiment to find the best model, which could affect the accuracy of flood vulnerability forecasting. Figure 7 shows also that all of the models have similar values from the measured data and predicted data. Further analysis showed that the high accuracy achieved by three models to decide the vulnerability of flood in Bandung (shown in Figure 8) is based on Equation (10).

In order to evaluate the performance of the proposed model, other commonly used techniques such as the Mamdani model and the Sugeno model were employed for comparison purposes. The forecasting errors obtained are presented in Table 9. For MAE measurement, the average error is 1.7797%, 1.4035%, and 1.2556% for the Mamdani model, the Sugeno model, and the proposed flood forecasting model (HN-FIS), respectively. These average values in RMSE measurement are 0.0382%, 1.9479%, and 0.0371% for the Mamdani model, the Sugeno model, and the proposed model (HN-FIS), respectively. The results showed that the proposed model reduces on flood vulnerability forecasting errors considerably in 31 subdistricts in Bandung, indicating the great improvement in flood vulnerability forecasting accuracy.

**Figure 9:**
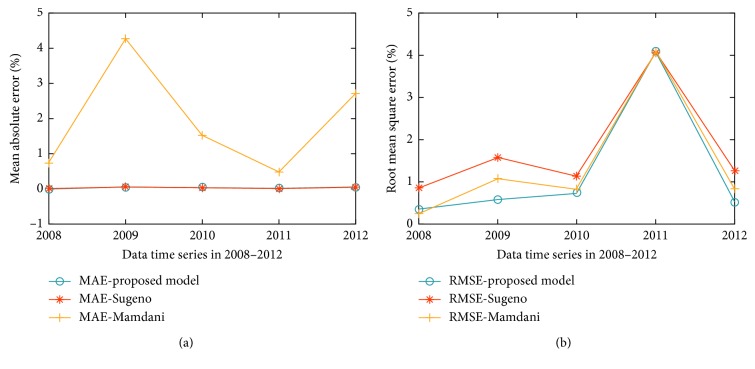
(a) MAE and (b) RMSE between Mamdani vs. Sugeno vs. proposed model (HN-FIS).

Figure 9 illustrates MAE and RMSE values in three models for flood vulnerability forecasting. The proposed model outperforms the other models and forecasted values correctly following the trend of flood variation.

Compared with the other methods presented in the literature using other databases, the proposed hybrid model provides reliable flood vulnerability forecasting, as shown in Table 9. In [35], the accuracy obtained in terms of MAE from 0.627 to 0.9357 and RMSE from 0.0523 to 0.1154 for flood prediction of 24 hours to 72 hours ahead of time. In [36], the accuracy of the average RMSE was 0.367% for flood forecasting in Tancheon Basin in Korea. The RMSE obtained with the proposed model varies from 0.0126% to 0.0548% for flood vulnerability forecasting. It means if an error is small, then accuracy will be close to real data and the model will give better flood vulnerability forecasting result. The proposed model achieved improvement in accuracy compared to the existing algorithms.

## 6. Conclusions

Neural network adopts a linear equation in the consequent part, which cannot present human assessment reasonably. In this case, we propose the hybrid model based on neural network and fuzzy inference system (HN-FIS) which has greater advantages in the following part and intuitive part of fuzzy reasoning. The proposed model has been constructed by a hybrid technique of the Mamdani model and Sugeno model based on the neurofuzzy inference system approach. The HN-FIS model can show its readability and understandability and present the essence of fuzzy logic more clearly. The current study aimed to determine and optimize the performance of the proposed model (HN-FIS). As supported by measurement and the predicted values based on simulation, the proposed model compares favorably with the Mamdani model and the Sugeno model in the capabilities of predicting the vulnerability of flood in 31 subdistricts in Bandung, Indonesia. The most apparent finding to emerge from this study is that three model flood forecasting (Mamdani, Sugeno, and proposed model) achieved the performance of more than 96%. However, the proposed model (HN-FIS) achieved the lowest error rate in both RMSE and MAE (0.0371% and 1.2556%, respectively) and obtained the best performance in flood vulnerability forecasting compared with existing models.

## Figures and Tables

**Table 1:** Rainfall in 31 subdistricts in Bandung.

Month	Rain precipitation (mm)				
	2008	2009	2010	2011	2012
January	265	208.5	353.3	63	82.9
February	166	200.5	557.1	76.7	303.7
March	425	365.7	531	89.4	155.5
April	342	165.6	93	381.5	290.8
May	132	183.8	345	193.4	257.1
June	20	101	191.9	117.6	60.5
July		24.2	220.8	77.2	34.2
August	80	0.5	220.8	3.1	0
September	45	24	424.4	102.8	27
October	303	234.5	292.2	103.6	125
November	455	318.2	401.4	321.4	537
December	333	271.1	237.5	259	637
**Rainfall/year**	**2566.0**	**2097.6**	**3868.4**	**1788.7**	**2510.7**

**Table 2:** Population density in 31 subdistricts in Bandung.

No.	Subdistrict	Population density (people/km^2^)				
		2008	2009	2010	2011	2012
1	Ciwidey	1613	1643	1465	1490	1500
2	Rancabali	355	359	324	330	332
3	Pasirjambu	342	346	334	339	341
4	Cimaung	1357	1377	1328	1355	1378
5	Pangalengan	734	748	710	723	728
6	Kertasari	463	466	432	428	430
7	Pacet	1139	1151	1100	1120	1129
8	Ibun	1391	1406	1389	1417	1428
9	Paseh	2026	2055	2053	2098	2118
10	Cikancung	1936	1954	2028	2084	2123
11	Cicalengka	3030	3093	3059	3123	3152
12	Nagreg	1005	1022	985	1008	1018
13	Rancaekek	3634	3691	3675	3760	3795
14	Majalaya	6284	6399	5976	6079	6125
15	Solokan Jeruk	3353	3390	3230	3289	3324
16	Ciparay	3266	3306	3270	3336	3369
17	Baleendah	4532	4602	5364	5580	5730
18	Arjasari	1421	1444	1401	1429	1447
19	Banjaran	2618	2638	2667	2726	2755
20	Cangkuang	2456	2479	2640	2743	2812
21	Pameungpeuk	4552	4591	4757	4876	4961
22	Katapang	4109	4193	4682	4866	4997
23	Soreang	2034	2075	2068	2123	2157
24	Kutawaringin	5963	6102	6126	6295	6374
25	Margaasih	7100	7245	7482	7728	7895
26	Margahayu	11655	11788	11417	11607	11687
27	Dayeuhkolot	10905	10993	10278	10388	10396
28	Bojongsoang	3071	3120	3804	3983	4133
29	Cileunyi	4211	4254	5197	5482	5709
30	Cilengkrang	1436	1458	1559	1613	1648
31	Cimenyan	1833	1871	1971	2031	2078

**Table 3:** Altitude of the areas in 31 subdistricts in Bandung.

No.	Subdistrict	Altitude of the area (masl)
1	Ciwidey	700–1200
2	Rancabali	1200–1550
3	Pasirjambu	1000–1200
4	Cimaung	765–1057
5	Pangalengan	984–1571
6	Kertasari	1250–1812
7	Pacet	700–1116
8	Ibun	700–1200
9	Paseh	600–800
10	Cikancung	600–1200
11	Cicalengka	667–850
12	Nagreg	715–948
13	Rancaekek	608–686
14	Majalaya	681–796
15	Solokan Jeruk	671–700
16	Ciparay	678–805
17	Baleendah	600–715
18	Arjasari	550–1000
19	Banjaran	750–800
20	Cangkuang	700–710
21	Pameungpeuk	650–675
22	Katapang	675–700
23	Soreang	700–825
24	Kutawaringin	500–1100
25	Margaasih	600
26	Margahayu	700
27	Dayeuhkolot	600
28	Bojongsoang	681–687
29	Cileunyi	600–700
30	Cilengkrang	600–1700
31	Cimenyan	750–1300

**Table 4:** Example of fuzzy sets in flood forecasting.

Population density	Altitude of the area	Rainfall	Vulnerability of flood
Very low	Low	Low	Safe
Low	Moderate	Moderate	Alert
High	High	High	Danger
Over		Extreme	

**Table 5:** Fuzzy classification of population density.

Population density rating	Very low	Low	High	Over
Population density (people/km^2^)	<350	[350, 3350]	[3500, 9000]	>9000

**Table 6:** Fuzzy classification of the altitude of the area.

Altitude of area rating	Low	Moderate	High
Altitude of the area (masl)	<500	[500, 1000]	>1000

**Table 7:** Fuzzy classification of rainfall.

Rainfall rating	Low	Moderate	High	Extreme
Rainfall value (mm)	[0, 50]	[50, 100]	[100, 200]	>200

**Table 8:** Fuzzy classification of the vulnerability of flood.

Vulnerability of flood rating	Safe	Alert	Danger
Vulnerability of flood	<248	[248, 374]	>374

**Table 9:** MAE and RMSE of the Mamdani model, the Sugeno Model, and the proposed flood forecasting model (HN-FIS).

Forecasting model	MAE (%)					RMSE (%)				
	2008	2009	2010	2011	2012	2008	2009	2010	2011	2012
Mamdani	0.8496	1.5693	1.1420	4.0662	1.2714	0.0302	0.0628	0.0354	0.0154	0.0473
Sugeno	0.2425	1.0729	0.8213	4.0499	0.8307	0.7519	4.2681	1.5261	0.4739	2.7197
HN-FIS	0.3489	0.5825	0.7382	4.0950	0.5133	0.0126	0.0477	0.0548	0.0294	0.0410

## Data Availability

The data used to support the findings of this study are available from the corresponding author upon request.
